# Subtypes of nursing students' volunteer motivation in COVID-19: a latent profile analysis

**DOI:** 10.1186/s12912-024-01699-1

**Published:** 2024-01-19

**Authors:** Fupei He, Beilei Lin, Xueting Liu, Yongxia Mei, Wenna Wang, Zhenxiang Zhang, Mingxu Wang

**Affiliations:** 1 School of Nursing and Health, Zhengzhou University, Zhengzhou City, Henan Province China; 2 Academic of Medical Science, Zhengzhou University, Zhengzhou City, Henan Province China; 3 School of Public Health, Xi’an Jiaotong University, Xi’an City, Shanxi Province China

**Keywords:** Nursing students, Volunteer motivation, Latent profile analysis, Influencing factors

## Abstract

**Background:**

One factor that influences nursing students' decision to engage in volunteer activities is volunteer motivation. It is important to understand the motivations of nursing students to volunteer. However, the majority of current studies have concentrated on the present level of nursing students' motivation to volunteer.

**Objectives:**

To identify subgroups of nursing students' volunteer motivation and its influential factors.

**Methods:**

From January to February 2022, a cross-sectional online study was conducted, and 2569 nursing students from 10 provinces in China were recruited. Participants completed the General Information Questionnaire, Volunteer Functional Inventory (VFI), Perceived Stress Scale (PSS) and Perceived Social Support Scale (PSSS). We explore the categories and characteristics of volunteer motivation using latent profile analysis. Then, we determined factors that affect undergraduate nursing students' volunteer motivation using multinomial logistic regression.

**Results:**

The volunteer motivation score of the nursing students was 77.65 (15.22). The study found that volunteer motivation could be divided into three categories: low-low protective volunteer motivation group (9.3%), general-high career values volunteer motivation group (37.5%), and high volunteer motivation group (53.2%). Perceived social support scale score, perceived stress scale score, gender, and grade significantly influenced the volunteer motivation of nursing students in different categories (both *P* < 0.05). Women were more likely to have higher motivation to volunteer than men, and fourth-year nursing students were more likely to be in general-high career values volunteer motivation group.

**Conclusions:**

The study highlights the significant heterogeneity in volunteer motivation among nursing students. Higher volunteer motivation was associated with higher perceived social support and lower perceived stress. In addition, gender and academic year were significant influencing factors. Nursing educators should develop targeted volunteer management plans based on the typological characteristics of the population to motivate nursing students to volunteer and promote the development of individual physical and mental health and social well-being.

**Trial registration:**

The survey was approved by the Biomedical Ethics Committee of the Department of Medicine, Xi’an Jiaotong University (No. 2022–0006).

## Introduction

The emergence of COVID-19 led to its rapid spread in China and around the world [1]. It poses major health risks worldwide, causing concern. The pandemic has caused about a surge in demand for healthcare, prompting nations to assess how to allocate resources, including personnel [2]. Nursing students are stepping forward as volunteers to assist with this growing need [3, 4]. Nursing students with specialized knowledge and skills can enhance the resilience of the healthcare system by volunteering to actively combat the epidemic [5]. According to Dyson's survey in London, 7 out of 137 nursing students were volunteering at the time of the survey [6]. In Brunei, the prevalence of university nursing students volunteering was 75.4% [7].

Volunteering is the selfless act in which individuals provide assistance to those in need within the framework of an organization [8]. It is characterized by altruism, voluntary participation, public benefit, and free-of-charge services [9]. Volunteering has practical significance not only for the beneficiaries, but also for the volunteers themselves [10]. On the one hand, nursing, as a highly specialized discipline, plays an indispensable role in public health and health promotion. As a reserve of future nurses, nursing students possess medical knowledge and skills that enable them to provide basic medical advice and health guidance to the beneficiaries. This can encourage the adoption of a healthier lifestyle and prevent the occurrence of diseases; on the other hand, participation in voluntary service is conducive to the physical and mental health of the participants [11–13], while studies show that volunteering enhances mental resilience and empathy [14–16]. Additionally, research on people over 50 has shown that volunteering can decrease mortality and enhance psychosocial well-being [17]. Therefore, promoting volunteering among nursing students holds significant importance.

Despite the general benefits of nursing students' participation in volunteering has been shown to have general benefits, such as promoting their overall health and improving their physical and mental well-being [18], studies have found that the actual involvement of nursing students in volunteering varies. Motivation for volunteering is related to volunteering behavior [9]. It has been suggested that motivation has a greater predictive effect on an individuals’ decision to participate in volunteering and continue to volunteer [19]. Volunteer motivation pertains to the internal drive of volunteers to perform volunteer service based on their need, recognition, or interest in the service itself [20]. During the COVID-19 pandemic, there is an expectation of increased volunteerism from nursing students [7, 21]. This is due to their intrinsic motivation driven by a strong sense of altruism [3, 22, 23]. Others have noted that volunteers were motivated by concerns about receiving lower grades, being excluded from future research opportunities, and even a perceived sense of coercion [24].

According to resource conservation theory [25], beneficial social support, positive psychological qualities, and environmental factors are considered valuable resources. Individuals with abundant resources are likely to have more enthusiasm and energy to motivate them to adopt more positive behaviors. Therefore, individuals' perceived social support and environmental pressure may influence their motivation to volunteer and their volunteer behavior. Gu et al. found that nurses had a moderate level of motivation to volunteer during the COVID-19 pandemic,which was negatively correlated with their perceived stress levels [26]. Previous studies have shown that the social support is a motivating factor for medical students to volunteer [3, 27]. Additionally, nursing students have reported that support and encouragement from family or the community had a positively impacted on their volunteering activities [23]. Furthermore, Amal identified several factors that affect the motivational willingness of nursing students to volunteer, including marital status, grade level, altruism, personal safety, and knowledge level [7]. A cross-sectional study conducted during an infectious disease pandemic in Saudi Arabia also revealed that gender, age, and grade level were significant motivational factors that influenced undergraduate nursing students' willingness to volunteer [28].

A systematic review found that health and nursing students' motivations for volunteering included domain value, understanding, enhancement, career, incentive, government, social, and demographics [29]. This motivation is explained by Clary & Snyder, namely increasing values such as altruism, learning and experience opportunities, personal growth and development, career-related clinical skills and experiences, and strengthening social relationships [30]. Therefore, nursing students' motivation for volunteering varies [19, 31], and there is a significant degree of heterogeneity [29]. There is a need to explore in-depth analysis of nursing students' motivations for volunteering. However, the majority of current studies have concentrated on the present level of nursing students' motivation to volunteer [7, 32]. Latent profile analysis (LPA) [33] is an individual-centered analytical technique that can improve differentiation among group categories by elucidating response characteristics and the proportion of individuals on various entries or dimensions. Therefore, this study aims to examine the possible categories of epidemic volunteer motivation among nursing students in colleges and universities and the variations in their characteristics via latent profile analysis. This will offer guidance for colleges, universities, and related departments in formulating practical and scientific volunteer service management programs, encouraging student participation in volunteer activities, and enhancing social health and well-being.

## Methods

### Design

From January to February 2022, a cross-sectional online study was conducted. The design and reporting of this study were conducted in accordance with the guidelines for Strengthening the Reporting of Observational Studies in Epidemiology (STROBE).

### Participants

An online anonymous cross-sectional survey of 2666 university nursing students in 10 Chinese provinces (including Tianjin, Henan, Hebei, Sichuan, Shaanxi, Shanxi, Shandong, Liaoning, Heilongjiang, and Guizhou) was conducted from January to February 2022 by using a convenience sample using Wenjuanxing. The inclusion criteria for participants were as follows: (1) being at least 16 years old and enrolled as a full-time student in China; (2) have consented to give informed consent and participate in this study. The exclusion criteria were as follows: (1) the response time of the online questionnaire was less than 180 s; (2) single answer time less than 2 s; (3) highly repetitive answers. A final total of 2569 valid questionnaires were returned, with a valid return rate of 96.36%.

### Sample size

LPA requires a sample size greater than 500 [34], so the sample size for this study is at least 500.

### Data collection tools

The questionnaire includes sociodemographic information. There were 8 questions, including age, gender, political appearance, ethnicity, grade, place of origin, education level, and student leader experience.

#### Volunteer functional inventory

Volunteer motivations were measured through the Volunteer Functional Inventory (VFI) [35]. The VFI is a voluntary functioning questionnaire developed by Clary et al. The questionnaire has 18 items and includes 6 dimensions: values (related to altruistic beliefs), understanding (learning new skills and exercising knowledge and abilities through volunteering), social (volunteering as an opportunity to have relationships with others and to conform to normative influences), career (career-related benefits from volunteering), protective (volunteering to protect the ego from negative problems), and enhancement (desire for personal growth and development through volunteering). A five-point Likert scale ranging from one (very disagree) to five (very agree) was used for each item. The scale scores range from 18–90, and the higher the score is, the higher the level of motivation of the respondent to volunteer. In this study, Cronbach's alpha for the VFI was 0.981. This study performed latent profile analysis on the total scores for each dimension in the questionnaire.

#### Perceived stress scale

The Perceived Stress Scale (PSS-10), developed by Cohen in 1983 [36], is a widely used tool for assessing perceived stress. It is mainly used to measure the level of stress in an individual's life. The original version included three dimensions of perceived loss of control, perceived unpredictability and perceived tension, with a total of 14 items, also known as the PSS-14; it was later adapted by Chaaya [37] et al. into the 10-item PSS-10, including two dimensions of perceived crisis and perceived ability to cope. A five-point scale was used to assign a positive score to the crisis perception dimension, from "none" to "most of the time", with scores ranging from 0 to 4, and a negative score to the coping ability dimension, which is the opposite. The total score ranges from 0 to 40, and the higher the score is, the higher the perceived stress. In this survey, the Cronbach's alpha coefficient for this scale was 0.878.

#### Perceived social support scale

The Perceived Social Support Scale (PSSS) [38] was developed by Blumenthal et al. to measure the extent to which individuals apprehend support from various sources of social support. The questionnaire consists of 12 items divided into three dimensions, with items 3, 4, 8, and 11 being family support, items 6, 7, 9, and 12 being friend support, and items 1, 2, 5, and 10 being other support. The questionnaire uses a 7-point scale, from "strongly disagree" to "strongly agree", with a score of 1 to 7, respectively, and the sum of the scores of each item is the total score. In this study, the Cronbach's alpha coefficient of the scale was 0.973.

### Data collection

Data was collected between January and February 2022. To recruit the study participants, the project leader contacted the director of the nursing school of 20 different levels of schools across the country and distributed the web-based questionnaire to the students through the director. The survey was conducted using Wenjuanxing (www.wjx.cn), a popular online data collection tool in China. The social media platform WeChat was used to distribute both the QR code and the link to the online questionnaire. The questionnaire began with a section explaining the purpose, meaning, and instructions for completion the survey before the formal questions. To avoid duplication, each IP address could only be entered once. There was no time limit for completing the questionnaire; but all items had to be completed before submission. Deleting single answer time less than 2 s and total answer time less than 180 s and regular answer type questionnaire, a total of 2,569 nursing students completed the survey, resulting in an effective response rate of 96.36%. The survey was approved by the Biomedical Ethics Committee of the Department of Medicine, Xi'an Jiaotong University (No. 2022–0006). Before participating in the survey, each participant voluntarily signed an online informed consent form.

### Quality control

Data was collected using Wenjuanxing. The same IP address answer permission only once. Respondents were required to answer all questions before submitting the questionnaire. After completing the survey, the data was double-checked and respondents who were not aged between 16–30 years old were excluded. Additionally, deleting single answer time less than 2 s and total answer time less than 180 s and regular answer type questionnaire.

### Data analysis

Data were analyzed using SPSS 26.0 and Mplus 8.3. The data analysis consisted of three parts. First, latent profile analysis was used to identify categories of volunteer motivation. The results of the 6 dimensions of the Volunteer Functional Inventory served as exogenous variables since the scale scores were continuous variables, and latent profile analysis was performed using the Mplus 8.3 program to separate the volunteer motivation of nursing students into different subgroups. Latent profile analysis (LPA) is a methodological approach that helps to explain population heterogeneity within observed data through the identification of underlying subgroups of individuals. Six model fit indexes were used to assist in determining the best model for LPA: Akaike Information Criterion (AIC), Bayesian Information Criterion (BIC), adjusted Bayesian Information Criterion (aBIC), Lo-Mendell-Rubin (LMR), Bootstrapped Likelihood Ratio Test (BLRT), and Entropy. For AIC, BIC, and aBIC, lower values indicated a better-fitting model. The aLMRT and BLRT statistical significance indicated that the model with the higher number of profiles was better than that with the lower number of profiles [34]. LMR and BLRT are used to make a comparison between the estimated model and a model with k-1 class, or classes, with k equaling the number of classes. A low and significant *P* value for the LMR and BLRT indicates that the estimated model outperforms the model with one fewer class. The entropy value evaluated the classification accuracy and ranged from 0 to 1, and a higher value represents the more accurate classification. And 0.8 entropy value is great, representing that more than 90% of families were classified into profiles accurately. The smallest proportion of profile cut-off was 0.05 [39].

SPSS 26.0 was then used for descriptive statistical analysis. Frequencies and proportions were used to describe categorical variables, while means and standard deviations were used to describe continuous variables if they passed the normality test. The chi-squared test was used to compare categorical variables between groups. Continuous variables were compared between groups using ANOVA.

Finally, multinomial logistic regression was used to analyze the factors influencing the categories of volunteer motivation of college and university nursing students.

## Results

### Nursing students' volunteer motivation, perceived social support and perceived stress scores

The mean scores of volunteer motivation (79.75 ± 10.69), perceived social support (62.95 ± 13.63), and perceived stress (30.22 ± 5.79) were obtained by nursing students in higher education. The specific scores of each dimension are shown in Table 1.

**Table 1:** Nursing students' VFI, PSSS and PSS-10 scores

Scale	M(SD)
Volunteer motivation	79.75(10.69)
Understanding	13.63(1.96)
Career	13.32(2.02)
Values	13.31(1.92)
Enhancement	13.34(2.06)
Protective	12.94(2.38)
Social	13.20(2.06)
Perceived Stress	30.22(5.79)
Perceived Social Support	62.95(13.63)

### Latent profile analysis of volunteer motivation in nursing students

We selected 1–5 latent profile models to analyze nursing students’ volunteer motivation. The results showed that the AIC, BIC, and aBIC values decreased as the number of categories increased. Two indicators, LMR and BLRT (*p* < 0.001), indicated that the models fit well for Classes 2 and 3. The value of entropy is closest to 1 for Class 3. A classification model of three latent categories (C1, C2, C3) was finally chosen to classify the nursing students’ volunteer motivation. As shown in Table 2.

**Table 2:** Latent profile model indexes of fitting for volunteer motivation types

Model	Log(L)^a^	AIC^b^	BIC^c^	aBIC^d^	Entropy	LMR^e^	BLRT^f^	Class probability(%)
1	-33016.424	66056.849	66127.064	66088.937	-	-	-	1
2	-27951.140	55940.280	56051.454	55991.086	0.966	< 0.001	< 0.001	44.113/55.887
3	-26570.607	53193.213	53345.347	53262.737	0.967	< 0.001	< 0.001	9.070/37.680/53.250
4	-25931.392	51928.784	52121.876	52017.026	0.973	0.057	< 0.001	8.047/37.286/53.888/0.780
5	-24512.734	49105.468	49339.519	49212.428	0.987	0.200	< 0.001	33.245/4.462/9.781/51.657/0.856

^a^Log likelihood

^b^Akaike information criterion

^c^Bayesian information criterion

^d^Adjusted bayesian information criterion

^e^Lo-Mendell-Rubin Likelihood Ratio Test

^f^Bootstrapped Likelihood Ratio Test

**Fig. 1:**
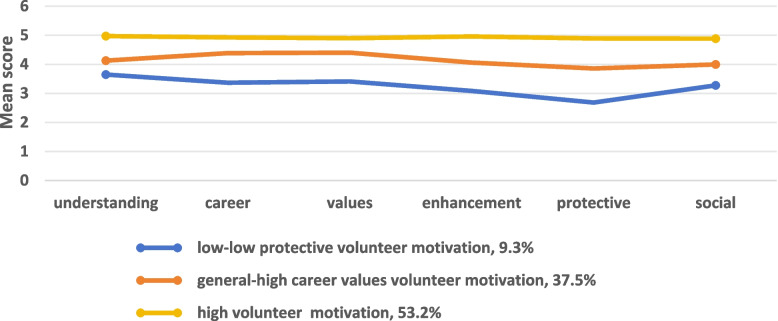
Profile of latent categories of volunteer motivation

Three latent categories differed obviously in their scores for the six items of volunteer motivation and showed different characteristics. C1 accounted for approximately 9.3% of the total number of subjects, and its score on each item was significantly lower than that of C2 and C3. Therefore, this category was named “low-low protective volunteer motivation”. C2 accounted for approximately 37.5% of the total, and its score on each item was higher than C1 but lower than C3 and was therefore named “general-high career values volunteer motivation”. C3 accounted for 53.2% of the total, and its score was significantly higher than that of C1 and C2. This category was therefore named “high volunteer motivation”. As shown in Fig. 1.

### Univariate analysis of latent categories of nursing students' volunteer motivation

As shown in Table 3, a total of 2569 valid questionnaires were collected in this survey. The results of the univariate analysis showed statistically significant differences in gender, grade, place of origin, whether or not they were student leaders, perceived stress scores, and total perceived social support scores (*P* < 0.05). In other general data, the difference was not statistically significant (*P* > 0.05).

**Table 3:** Demographic and characteristics by latent profile (*N* = 2569)

**Variables**	**Class 1**	**Class 2**	**Class 3**	**χ2**/***F***	***p***
Age, Mean (SD)	19.76 (1.64)	19.61 (1.43)	19.59 (1.45)	1.293	0.275
Gender				20.247	< 0.001
Male	58 (2.26%)	236 (9.19%)	445 (17.32%)		
Female	175 (6.81%)	732 (28.49%)	923 (35.93%)		
Ethnicity				3.416	0.181
Han	224 (8.72%)	950 (36.98%)	1337 (52.04%)		
Ethnic Minority	9 (0.35%)	18 (0.70%)	31 (1.21%)		
Grade				26.123	< 0.001
First Grade	118 (4.59%)	503 (19.58%)	824 (32.07%)		
Second Grade	94 (3.66%)	397 (15.45%)	454 (17.67%)		
Third Grade	17 (0.66%)	54 (2.10%)	76 (2.96%)		
Forth Grade	2 (0.08%)	10 (0.39%)	4 (0.16%)		
Fifth Grade	2 (0.08%)	4 (0.16%)	10 (0.39%)		
Place of origin					
Urban	58 (2.26%)	220 (8.56%)	378 (14.71%)	7.226	0.027
Rural	175 (6.81%)	748 (29.12%)	990 (38.54%)		
Education level				10.723	0.097
Junior college	167 (6.50%)	763 (29.70%)	1004 (39.08%)		
Bachelor’s	51 (1.99%)	161 (6.27%)	205 (7.98%)		
Master’s	10 (0.39%)	27 (1.05%)	54 (2.10%)		
Doctor’s	5 (0.19%)	17 (0.66%)	15 (0.58%)		
Political Appearance				14.718	0.065
Member of the Communist Party of China	6 (0.23%)	4 (0.16%)	16 (0.62%)		
Preparatory member of the Communist Party of China	9 (0.35%)	55 (2.14%)	78 (3.04%)		
Member of the Communist Youth League	165 (6.42%)	668 (26.00%)	899 (34.99%)		
General public	52 (2.02%)	238 (9.26%)	371 (14.44%)		
Others	1 (0.04%)	3 (0.12%)	4 (0.16%)		
Student leaders				6.169	0.046
Yes	65 (2.53%)	274 (10.67%)	448 (17.44%)		
No	168 (6.54%)	694 (27.01%)	920 (35.81%)		
Perceived Stress, Mean (SD)	30.74 (*4.40*)	30.46 (*4.21*)	29.96 (*6.86*)	3.118	0.044
Perceived Social Support, Mean (SD)	52.64 (*12.14*)	59.11 (*10.59*)	67.43 (*14.00*)	207.742	< 0.001

C1, low-low protective volunteer motivation

C2, general-high career values volunteer motivation

C3, high volunteer motivation

### Multiple logistic regression analysis of latent categories of nursing students' volunteer motivation

Multiple logistic regression analysis was performed with three variables, C1 low-low protective volunteer motivation, C2 general-high career values volunteer motivation and C3 high volunteer motivation, as the outcome variables and C3 group as the reference group. The results showed that the statistically significant indicators were gender, grade, perceived social support, and stress perception; women were more likely to have higher motivation to volunteer than men in the C1 and C3 groups; the higher the perceived social support score was, the stronger the motivation to volunteer; and the lower the stress perception score was, the higher the motivation to volunteer. Similarly, female students were more likely to be more motivated to volunteer than male students in groups C2 and C3, and the higher the perceived social support score was, the stronger the motivation to volunteer; the lower the stress perception score was, the higher the motivation to volunteer. In addition, fourth-year nursing students were more likely to be in group C2, as shown in Table 4.

**Table 4:** Multinomial logistic regression analysis of potential categories of medical students' volunteer motivation

	**C1 VS.C3**							**C2 VS.C3**						
**Categories**	***β***	**SE**	**Waldχ**^**2**^	***P***	***OR***	**95%*CI***		***β***	**SE**	**Waldχ**^**2**^	***P***	***OR***	**95%*CI***	
						**LLCI**	**ULCI**						**LLCI**	**ULCI**
Gender(Ref:male)														
Female	-0.382	0.175	4.764	0.029**	0.683	0.484	0.962	-0.348	0.101	11.867	< 0.001**	0.706	0.579	0.861
Grade(Ref:Fifth Grade)														
First Grade	0.250	0.874	0.082	0.775	1.283	0.231	7.117	0.678	0.628	1.165	0.280	1.969	0.575	6.744
Second Grade	0.458	0.876	0.274	0.601	1.581	0.284	8.794	0.952	0.629	2.288	0.130	2.590	0.755	8.891
Third Grade	0.390	0.913	0.182	0.669	1.477	0.247	8.835	0.665	0.652	1.038	0.308	1.944	0.541	6.982
Forth Grade	0.999	1.261	0.628	0.428	2.715	0.229	32.122	1.798	0.873	4.244	0.039*	6.036	1.091	33.383
Place of origin(Ref:Rural)														
Urban	-0.037	0.174	0.045	0.832	0.964	0.686	1.355	-0.189	0.103	3.350	0.067	0.828	0.676	1.013
Student leaders(Ref:No)														
Yes	-0.199	0.168	1.395	0.237	0.820	0.590	1.140	-0.159	0.098	2.632	0.105	0.853	0.704	1.034
Perceived Stress	0.044	0.015	8.748	0.003**	1.045	1.015	1.076	0.019	0.008	5.513	0.019*	1.019	1.003	1.036
Perceived Social Support	-0.093	0.006	206.277	< 0.001**	0.911	0.899	0.923	-0.050	0.004	199.049	< 0.001**	0.951	0.944	0.958

C1, low-low protective volunteer motivation

C2, general-high career values volunteer motivation

C3, high volunteer motivation

^*^p < 0.05

^**^p < 0.01

## Discussion

The survey results indicate that nursing students in colleges and universities exhibit a high level of motivation for volunteering, with over half of them expressing a strong motivation to volunteer. Consistent with prior research, the majority of nursing students were willing to participate in volunteer activities, driven by a sense of responsibility and mission [40, 41]. We found that the protective dimension was lower in the C1 group than in the other dimensions of the group, This suggests that when motivating nursing students to volunteer, the use of self-protection dimensions, such as the ability to improve loneliness and forget about annoying things, should be carefully considered.

Our study found that the C2 group scored higher on the dimensions of values and career development compared to others dimensions, indicating that some nursing students were more willing to volunteer out of a desire to practice altruism and humanitarianism, as well as being motivated to volunteer to promote their future career development. Volunteers who are highly motivated to volunteer by altruistic and humanitarian values (VFI values) were more likely to select volunteering activities involving patient contact [23]. A systematic review found that the VFI subscales with the highest scores among medical and nursing volunteers in healthcare settings were VFI Values and Enhancement [42]. A values-centric approach may be common among nursing students who volunteer with patients. Clary et al. found that recruitment appeals are most effective when matched with the internal state of volunteers, and Kpanake et al. concluded that broad value-based motivational messaging would be the best way to attract medical volunteers in epidemic response [35, 43]. Therefore, orienting nursing students towards values-centric motivations may increase their participation in patient contact activities.

The study results indicate that perceived social support is a predictor of higher motivation to volunteer. Improving nursing students' perceived social support can stimulate their motivation to volunteer. This finding is consistent with previous research, where perceived social support was positively associated with medical students' motivation [44]. Furthermore, a qualitative study on nursing students' volunteering behaviours found that their motivation for volunteering was influenced by their perception of social support [45]. The Pygmalion Effect suggests that social support can have a positive impact on people's behaviour [46]. When individuals engage in pro-social activities, such as volunteering, they are more likely to receive social recognition and praise. Social support can enhance an individual's self-worth and promote pro-social behaviour, leading to positive outcomes. The conservation of resources theory suggests that positive psychological quality, helpful social support, and contextual factors are considered to be valuable resources [25]. Individuals with abundant resources may have more enthusiasm and energy to engage in positive behaviors [47, 48]. Beneficial social support motivates individuals to be enthusiastic enough to engage in more positive behaviors, such as volunteering. These studies provide a theoretical reference to reveal the impact of perceived social support on voluntary behavior. Therefore, in promoting nursing students' participation in volunteering activities, it is possible to mobilize their surrounding family and friends to increase individuals' perceived social support and motivation to volunteer. It is suggested that volunteer organization managers and educators should pay attention to creating a positive social supportive environment. Society, schools and healthcare organizations should give multi-level social support to nursing students, such as parents and educators should give them prompt emotional support and guidance on volunteering to enhance students' sense of belonging and identity. Social and healthcare organizations should also detail the volunteering mechanism for nursing students, clarify the content of volunteering and provide targeted training.

Furthermore, this study demonstrates that the perceived stress levels of nursing students in college have an impact on their motivation to volunteer. Specifically, and higher levels of stress are associated with lower levels of motivation to volunteer. This finding is consistent with previous research, which has shown that individuals with lower perceived stress are more likely to be motivated to volunteer [26]. Deci and Ryan [49] proposed self-determination theory, self-determination Theory suggests that human behavioral motivation results from the interaction of intrinsic needs and extrinsic environmental factors. These intrinsic needs include the pursuit of autonomy, competence, and relatedness. If external conditions do not support the fulfilment of these three fundamental psychological needs, then they do not support to internal motivation, and the individual's work attitude and behavioural performance are negatively affected to some extent [50]. Baard [51] et al. have shown that a work environment that meets an individual's need for autonomy, competence, and relatedness can enhance intrinsic motivation. In addition to the external environment, self-determination theory also emphasizes the impact of autonomy, competence, and belongingness on individual behavioral motivation. Individuals are most motivated to act when they have autonomy over their volunteering behavior and a sufficient sense of competence and belongingness. If individuals feel too much pressure and constraint in volunteering, their autonomy and sense of competence may be weakened, thus affecting their motivation to volunteer. Therefore, as individuals perceive that more stress is generated in their environment, internal motivation decreases, so as nursing students' stress perception scores continue to rise, motivation to volunteer decreases. Therefore, in the process of promoting nursing students' participation in volunteer activities, administrators and educators of volunteer organizations should pay attention to students' mental health and provide psychological support and assistance to reduce students' stress and improve their self-confidence and competence.

Our study found that women demonstrate higher motivation to engage in voluntary activities than men, supporting prior research [52]. This may be due to women being perceived as more approachable and attentive to interpersonal connections and emotional communication. Their tendency towards empathy and willingness to assist others may lead them to participate in volunteer work more often than men. In addition, there is a common perception that women are more socially responsible and compassionate than men. This can lead to a greater focus on resolving social issues and helping the disadvantaged, which may result in a higher likelihood of engaging in volunteer work. Women's inclination towards volunteering may also be influenced by their emotional and social needs. Volunteering provides a social platform for women to communicate and engage with others, while fulfilling their emotional needs, such as a sense of self-worth and satisfaction. Nursing students in their fourth year of college are relatively less motivated to volunteer, which may be related to the lack of time to volunteer when they are in their internship and graduation year, and several studies [28, 53] have shown that time is a strong influencing factor that prevents nursing students from volunteering. It is possible that senior academic students experience stress due to various factors such as clinical placements, employment, and career planning.

### Limitations

This study has the following limitations. First, although we aimed to investigate nursing students across the country, our study relied on a convenience sample and a self-reported web-based questionnaire, which may have biased the results. To increase the representativeness of the study sample in subsequent research, convenience sampling should be avoided. In addition, we did not compare score levels with other populations, such as non-nursing medical students, general college students, and clinical nurses. Finally, only nursing students from mainland China were included in the study, and the results may differ slightly from those of other countries. Therefore, more diverse populations, types, and rigorously designed studies are needed to confirm the findings in the future.

## Conclusions

This study used latent profile analysis to identify the category characteristics and their influencing factors of university nursing students' motivation for volunteering in the COVID-19. Nursing students' volunteering motivation can be categorized into three latent profiles: low-low protective motivation (9.3%), general-high career values motivation (37.5%), and high motivation (53.2%). Multinomial logistic regression results indicate that social support comprehension, stress perception scores, gender, and grade level influence different latent profiles. These findings provide new insights for management programmes and policy formulation regarding volunteerism among university nursing students during the epidemic.

## Data Availability

The datasets generated and/or analysed during the current study are not publicly available due [REASON WHY DATA ARE NOT PUBLIC] but are available from the corresponding author on reasonable request.
